# Proportional relationship between transcript concentrations and carbon biomass for open ocean plankton groups

**DOI:** 10.1093/ismejo/wraf079

**Published:** 2025-04-30

**Authors:** Sacha N Coesel, Shiri Graff van Creveld, Mathilde Dugenne, Fernanda Henderikx-Freitas, Angelicque E White, E Virginia Armbrust

**Affiliations:** School of Oceanography, University of Washington, Seattle, WA 98195, United States; School of Oceanography, University of Washington, Seattle, WA 98195, United States; Department of Oceanography, University of Hawaii at Manoa, Honolulu, HI 96822, United States; Department of Oceanography, University of Hawaii at Manoa, Honolulu, HI 96822, United States; Department of Oceanography, University of Hawaii at Manoa, Honolulu, HI 96822, United States; School of Oceanography, University of Washington, Seattle, WA 98195, United States

**Keywords:** metatranscriptomics, spike-in, carbon biomass, protist, phytoplankton, dinoflagellate, north pacific ocean, transcript concentration, imaging FlowCytobot, transcript-to-biomass relationship

## Abstract

Unicellular plankton form the foundation of the marine food web, driving carbon fixation and cycling essential biogeochemical elements in marine ecosystems. Carbon biomass, often measured as a bulk property, serves as a common “currency” for ecologists. The increasing availability of metatranscriptomic data presents an opportunity to add taxonomic and functional resolution to ecological models and yet, aligning transcript counts with carbon biomass estimates remains a challenge. Here, we combine 30 quantitative metatranscriptome samples with Imaging FlowCytobot-derived carbon biomass estimates and demonstrate a robust, proportional (log–log scale) relationship between transcript concentration and carbon biomass estimates across abundant protist taxa. Further, we show that dinoflagellates exhibit a transcript-to-biomass ratio ~ 6.4-fold higher than other protist groups, consistent with their known transcriptional divergence. These findings provide a means to address overrepresentation of dinoflagellate transcript levels in metatranscriptome data. Moreover, this study establishes an entrée for integrating metatranscriptomics into carbon biomass-based ecological models, enhancing the interpretability and applicability of transcriptomic data in ecosystem research and modeling.

Metatranscriptome sequencing of seawater samples provides a window into planktonic community structure and function. Use of spiked-in synthetic standards of known concentration allows the calculation of transcript concentrations of taxonomic plankton groups in units of transcripts per taxon per liter [1]. Whereas macromolecular cell components broadly scale with cell size [2], the relationship between metatranscriptome transcript counts and taxonomic abundance remains unclear. Gene expression levels can exhibit fluctuations at both the population and individual cell levels [3], yet the impact on transcript-to-biomass ratios is unknown. Also, ubiquitous and abundant taxa such as Dinoflagellata may produce more RNA molecules per biomass unit due to their distinct transcriptional regulation [4].

Here, we combined quantitative metatranscriptomics with ﻿Imaging FlowCytobot (IFCb)-based carbon biomass estimates. The IFCb is an automated imaging flow cytometer that captures high-resolution cell images, enabling machine-learning-based quantification of cell abundance, biovolume, and taxonomy [5]. Our data were derived from two ~2000 km surface transects (Gradients 2 and 3) spanning ∼25°-41° N along 158° W in the Pacific Ocean (Fig. S1), a region dominated by picocyanobacteria, as well as picoeukaryotes and nano-sized protists, particularly Dinoflagellata [6, 7]. We calculated mean estimated carbon (C) biomass and transcript concentrations from 36 quantitative poly-A-selected metatranscriptomes [8] for five abundant eukaryotic taxonomic groups (Haptophyta, Bacillariophyta, Dictyochophyceae, Dinoflagellata, and Ciliophora) within the ∼3–100 μm cell size range. We found a proportional relationship between log-transformed transcript abundance and carbon biomass concentration, enabling comparisons of transcript-to-biomass relationships across taxonomic groups.

**Figure 1:**
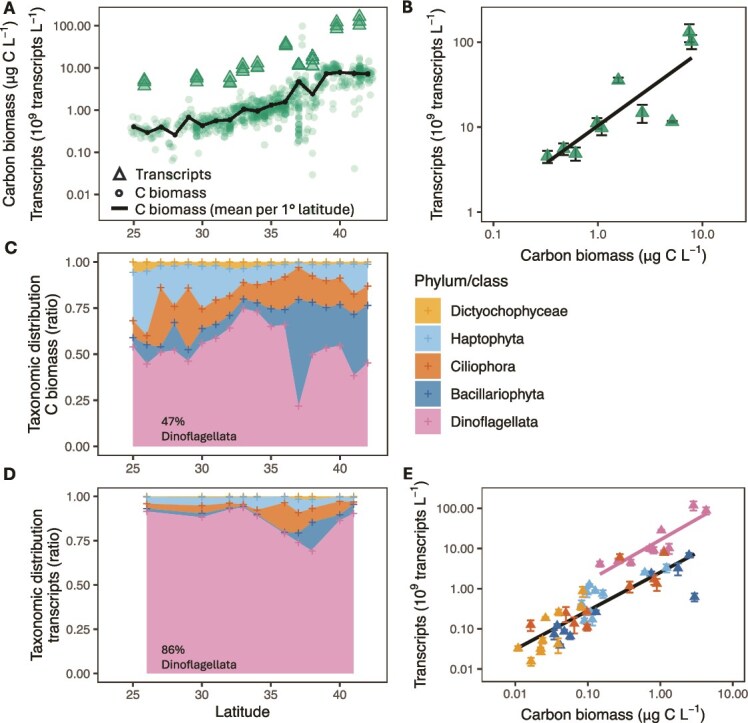
Comparison of functional mRNA transcript concentrations and IFCb-estimated C biomass concentrations of combined Dinoflagellata, Bacillariophyta, Ciliophora, Haptophyta and Dictyochophyceae within the 3–100 μm size class. Samples were collected along the 158°W surface transect of the North Pacific Ocean during the 2017 scope gradients 2 cruise. (A) IFCb-estimated C biomass in μg carbon per liter per sample (circles) and mean IFCb C biomass per 1° latitude (solid line). Functional (KOfam)-annotated mRNA transcripts in 10^9^ transcripts per liter (triangles). (B) Linear regression (solid line) of log10-transformed functional-annotated transcripts versus log10-transformed IFCb-estimated C biomass (slope = 0.89; *R*^2^ = 0.72). (C) Relative taxonomic distribution of the C biomass per 1° latitude. Crosses represent aggregation by 1° latitude. (D) Relative taxonomic distribution of the transcript concentrations per sampling station, crosses represent sampling location. (E) Linear regressions of log-transformed functional-annotated transcript versus log-transformed IFCb-estimated C biomass for Dinoflagellata (pink; slope = 1.03; *R*^2^ = 0.78), and the other combined taxonomies (black; slope = 0.96; *R*^2^ = 0.73). Samples are indicated by the triangles. Error bars indicate the standard deviation of biological triplicates.

Metatranscriptome mixed surface layer samples were collected during Gradients 2 (5/27/2017–6/13/2017) at 15 m depth using a CTD rosette, and during Gradients 3 (10/4/2019–10/29/2019) at ~7 m depth using the ship’s underway intake system. The processed metatranscriptomes are published in the North Pacific Eukaryotic Gene Catalog [8]. Briefly, samples were pre-filtered using a 100 μm mesh and collected by filtration on 3 μm polycarbonate filters [8]. Filters were flash-frozen in liquid nitrogen and stored in −80°C until RNA extraction with Direct-zol RNA MiniPrep Plus kit (Zymo Research) [8]. ﻿Prior to RNA extraction, samples were spiked with a set of synthetic RNA standards to quantify extraction/sequencing efficiency. [1, 8]. ﻿Poly-A-selected mRNA was sequenced on the NovaSeq 6000 System (Illumina). Standard-derived normalization factors were obtained for each sample as follows:


$$ Normalization\ factor=\frac{Standards_{added}}{Standards_{retrieved}}\div{Volume}_{sample.} $$

Quality-controlled read pairs were ﻿assembled de novo *﻿*using Trinity [9], and taxonomically and functionally annotated using MarFERReT v1.1 [10] and KofamScan [11], respectively [8]. Contigs with at least 10 mapped transcripts were maintained. Transcript concentrations (in 10^9^ transcripts L^−1^) of Dinoflagellata, Bacillariophyta, Ciliophora, Haptophyta, and Dictyochophyceae were calculated per sample by applying the standard-derived normalization factors to mRNAs associated with a KOfam functional annotation [11] (Fig. 1), or to total mRNA pools (Fig. S1), which include mRNA of unknown function.

As with the metatranscriptome samples, the IFCb data were collected from within the mixed surface layer using the ship’s underway intake system at ~7 m depth [6]. IFCb data from Gradients 2 were collected continuously throughout the transect, Gradients 3 data were restricted to discrete stations. IFCb scattering and fluorescence gains were set to 0.4 and 0.5 for Gradients 2, and 0.8 and 0.87 for Gradients 3, respectively [6]. Particles <100 μm in diameter were classified by machine-learning based on morphology [5, 6]. Cells near the detection limit (3–5 μm diameter) may be underestimated. Biovolumes were calculated from 2D images [6] and converted to cellular C biomass via allometric scaling (Table S1) [12]. The cellular carbon quotas of Dinoflagellata, Bacillariophyta, Ciliophora, Haptophyta, and Dictyochophyceae were summed per sampling volume, converted to μg C L^−1^, and averaged per 1° latitude (Fig. 1A).

The transcript and C biomass concentrations across the Gradients 2 transect followed broadly intercomparable patterns, both spanning over an order of magnitude from 25°N to 41°N (Fig. 1A). Transcript concentrations were compared to the mean C biomass concentrations at the nearest 1° latitude intervals, resulting in 10 matched locations (Fig. 1B). Akaike Information Criterion (AIC) indicated the best linear model fit using log10-transformed carbon and transcriptome data (mean of biological triplicates; AIC = 7.7), compared to untransformed data (AIC = 95.9), or non-linear regression (AIC = 91.9). The transcript-to-biomass ratio (log10-transformed data) follows a power-law correlation (slope = 0.89; *R*^2^ = 0.72; Fig. 1B). Dinoflagellates accounted for approximately half of the estimated C biomass (Fig. 1C) and over 80% of functionally annotated transcripts (Fig. 1D) within the 3–100 μm size class. Analysis of Covariance (ANCOVA) indicated that carbon concentration was a significant predictor of transcript concentration (*P <* 0.001), and that taxonomy was a significant predictor of transcripts per unit carbon (*P* < 0.001). Dinoflagellata had significantly different transcript concentrations per unit carbon compared to all other taxa (Tukey post-hoc test, *P* < 0.05). The remaining taxa were not statistically different from each other and were considered a single group for further analysis.

A proportional correlation (log10-transformed data) was observed between transcript concentrations and IFCb-derived carbon biomass for both Dinoflagellata and the combined non-dinoflagellate taxonomies (Fig. 1E). The transcript-to-biomass ratio for Dinoflagellates was 6.4 (SE = 0.8) fold higher than the other plankton taxonomies (Table 1). This scaling factor remains consistent when using total mRNA transcript abundance per C biomass (Fig. S2 and Table 1). Analysis of the smaller Gradients 3 dataset also yielded proportional relationships for the broad taxonomic groups, with a Dinoflagellate scaling factor of ~6.4 (Fig. S3 and Table 1), indicating that this factor can be used to correct for the overestimation of Dinoflagellata in marine plankton metatranscriptome analyses. Our data span the North Pacific oligotrophic Subtropical Gyre and northern Transition Zone (Fig. S1). Whether a comparable relationship is observed in regions with higher biomass or during bloom conditions remains to be tested.

**Table 1:** Linear regression between transcript concentration (log10 T, 10^9^ transcripts L^−^^1^) and carbon biomass (log10 C, μg C L^−^^1^) for Gradients 2 and 3.

**Equation**	**Cruise**	**mRNA**	**Taxa**	**Intercept (*a*)**	**Slope (*b*)**	**N**	***R*** ^***2***^	**Dino-factor** ^**4**^
*T = aC^B^*	Gradients 2	functional^1^	Dinoflagellate	16.36 (1.38)	1.03 (0.20)	*10*	0.78	6.40 (0.87)
			non-Dinoflagellate^3^	2.56 (0.27)	0.96 (0.09)	*40*	0.73	
		total^2^	Dinoflagellate	42.82 (3.18)	*0.98 (1.72)*	*10*	0.80	6.45 (0.83)
			non-Dinoflagellate	6.64 (0.70)	0.90 (0.09)	*40*	0.71	
	Gradients 3	functional	Dinoflagellate	2.97 (NA)	1.44 (NA)	2	1.00	6.40 (NA)
			non-Dinoflagellate	0.46 (0.09)	0.98 (0.25)	*6*	0.72	
		total	Dinoflagellate	7.67 (NA)	1.13 (NA)	*2*	1.00	6.62 (NA)
			non-Dinoflagellate	1.16 (0.13)	1.03 (0.15)	*6*	0.89	

^1^Functional mRNA includes transcripts with KOfam annotation [11], whereas ^2^total mRNA includes all mapped transcripts. ^3^Non-Dinoflagellate taxonomies include Bacillariophyta, Ciliophora, Haptophyta, and Dictyochophyceae. Linear regressions were derived from log-transformed data using the mean of biological triplicates. The intercepts correspond to C = 1 μg C L^−1^. N represents the number of metatranscriptome sampling stations. Values in parentheses indicate standard error (SE) of estimates. For N = 2, SE is not available (NA). The Dinoflagellate scaling factor (^4^Dino-factor) is derived from the linear model predictions. IFCb scattering and fluorescence gain settings were 0.4 and 0.5 for Gradients 2, and 0.8 and 0.87 for Gradients 3.

Consistent patterns emerged that correlate carbon biomass with transcript levels across broad taxonomic groups and environmental conditions, despite variations in transcript abundance within individual cells. This suggests that plankton biomass can be estimated from quantitative metatranscriptome datasets. In the Gradients 2 dataset, the functional transcriptome averaged 2.56 * 10^9^ and 16.36 * 10^9^ transcripts per μg C for non-Dinoflagellate plankton and Dinoflagellata, respectively (Table 1). Absolute biomass estimates are sensitive to instrument settings, as indicated by significantly higher biomass estimates measured during the Gradients 3 cruise where higher scatter and fluorescence gain settings were used. These adjustments led to overall lower transcript-to-carbon ratios, yielding 0.46 * 10^9^ and 2.97 * 10^9^ functional transcripts per μg C for non-Dinoflagellate plankton and Dinoflagellata, respectively (Table 1). Similarly, quantitative metatranscriptome outcomes likely reflect variations in preservation, extraction, normalization, and bioinformatic pipelines, including reference database composition. Whereas these findings emphasize the importance of standardizing and calibrating measurements in future studies, the observed range aligns with single-cell total mRNA sequencing estimates (Table S2) [13], illustrating the strength of environmentally derived data to uncover macromolecular relationships across diverse environmental conditions and population structures.

## Supplementary Material

Figures_SI_Coesel_04_21_2025_wraf079

## Data Availability

The code used for quantitative metatranscriptomics and the RNA extraction protocol is available at https://github.com/armbrustlab/NPac_euk_gene_catalog. The metatranscriptome sequences are available at the NCBI Sequence Read Archive (SRA) and the Transcriptome Shotgun Assembly (TSA) sequence database under BioProject PRJNA1076191 and PRJNA1076851. Transcript counts, taxonomic and functional annotations are available at https://zenodo.org/records/13826820 and https://zenodo.org/records/12630398. IFCb-derived datasets can be found at https://zenodo.org/records/4267135 and https://zenodo.org/records/4267140, and at the Simons Collaborative Marine Atlas Project (https://simonscmap.com).
