# Supplementary material for: Proportional relationship between transcript concentrations and carbon biomass for open ocean plankton groups
Source: ISME J. 2025 Apr 30;19(1):wraf079. doi: 10.1093/ismejo/wraf079 (PMC12085914; doi:10.1093/ismejo/wraf079)
Supplement: Figures_SI_Coesel_04_21_2025_wraf079 [file figures_si_coesel_04_21_2025_wraf079.pdf]

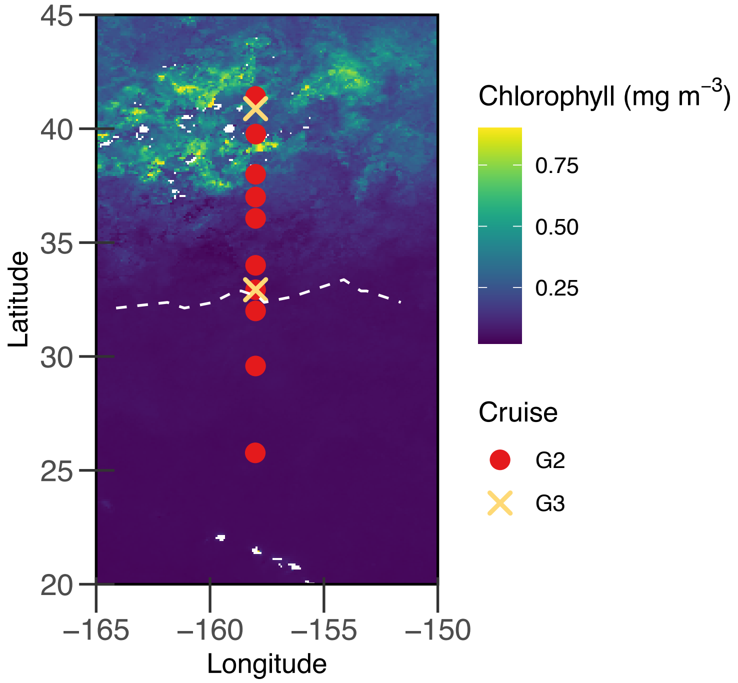

Figure S1. Metatranscriptome sampling locations of the Gradients 2 (G2; 2017; red circles) and Gradients 3 (G3; 2019; yellow crosses) cruises. Background color represents Chlorophyll concentration (MODIS Aqua satellite https://oceandata.sci.gsfc.nasa.gov, obtained via https://simonscmap.com, averaged over 05/01/2017 - 07/01/2017. The 34.82 surface isohaline, defining the boundary between the North Pacific Subtropical Gyre to the North Pacific Transition Zone as in Juranek, *et al.,* 2020 [6], is indicated by the dashed white line.

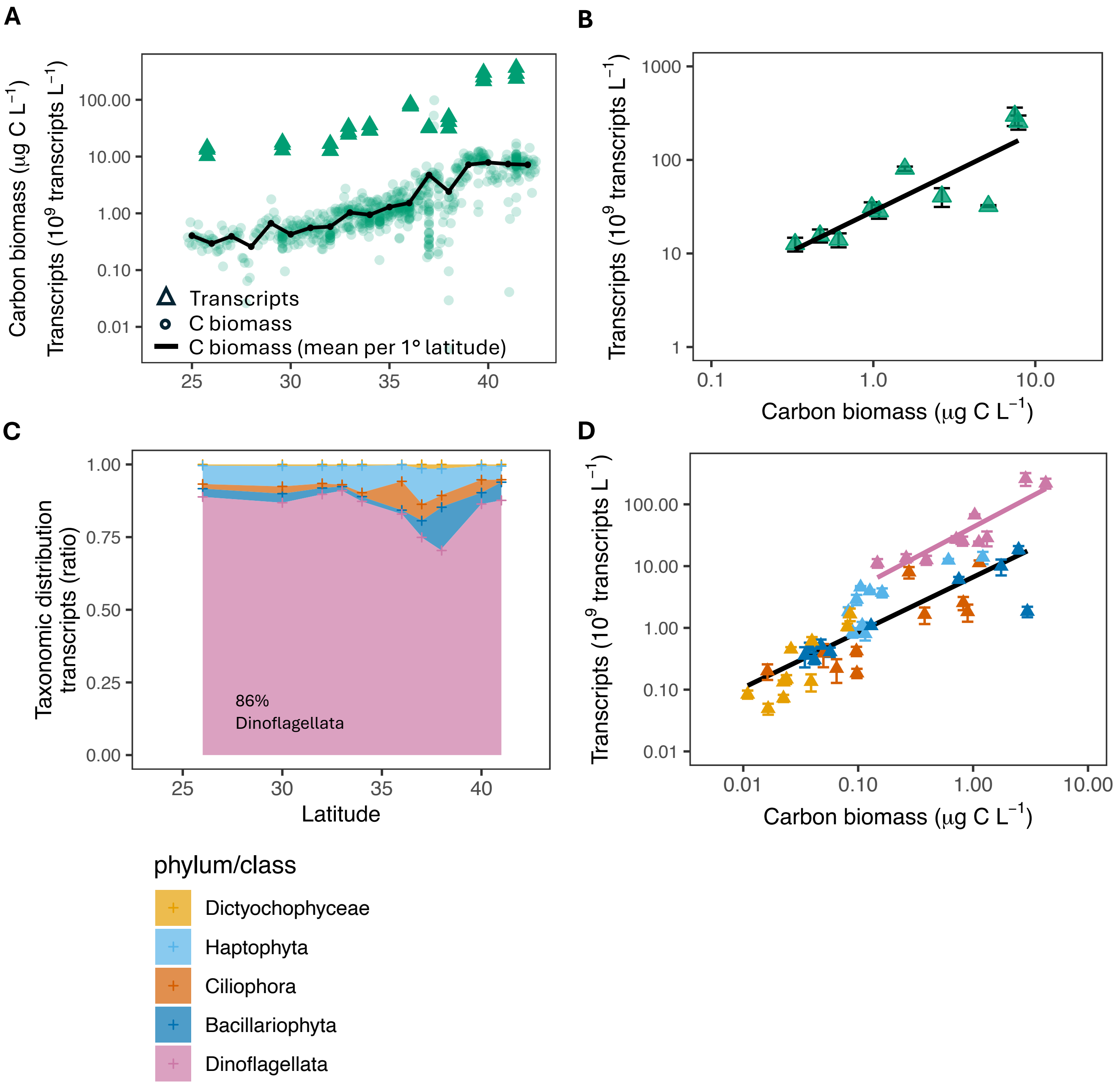

Figure S2. Comparison of total mRNA transcript concentrations and IFCb-estimated C biomass concentrations of combined Dinoflagellata, Bacillariophyta, Ciliophora, Haptophyta and Dictyochophyceae. Samples were collected along the 158°W surface transect of the North Pacific Ocean during the 2017 Scope Gradients 2 cruise. (A) IFCb-estimated C biomass in µg carbon per liter per sample (circles) and mean IFCb C biomass per 1° latitude (solid line). Total mRNA transcripts in 10^9 transcripts per liter (triangles). (B) Linear regression (solid line) of log10-transformed transcripts versus log10-transformed IFCb-estimated C biomass (slope = 0.84; R^2 = 0.74). (C) Relative taxonomic distribution of the C biomass per 1° latitude. Colors represent taxonomies, crosses represent aggregation by 1° latitude. (D) Relative taxonomic distribution of the functional transcript concentrations per sampling station, colors represent phyla, crosses represent sampling location. (E) Linear regressions of log-transformed transcript versus log-transformed IFCb-estimated C biomass for Dinoflagellata (pink; slope = 0.98; R^2 = 0.80), and the other combined taxonomies (black; slope = 0.90; R^2 = 0.71). Samples are indicated by triangles and colored as in C and D. Error bars indicate the standard deviation of biological triplicates.

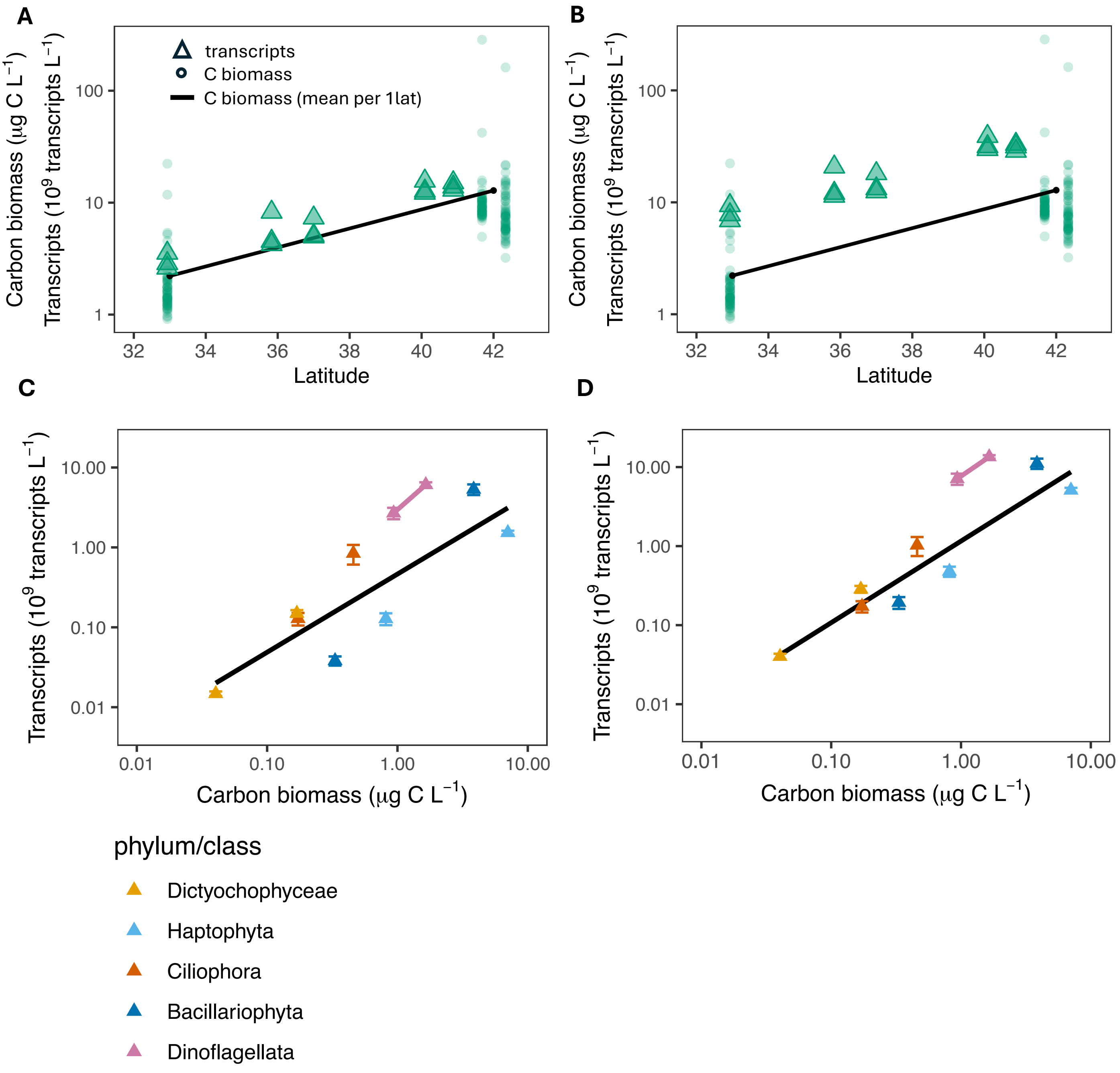

Figure S3. Comparison of (A) functional, and (B) total mRNA transcript concentrations and IFCb-estimated C biomass concentrations of Dinoflagellata, Bacillariophyta, Ciliophora, Haptophyta and Dictyochophyceae. Samples were collected along the 158°W surface transect of the Pacific Ocean during the 2019 Scope Gradients 3 cruise. A) The circles show the IFCb-estimated C biomass in µg carbon per liter per sample. The solid black line shows the mean IFCb C biomass per 1 degree latitude, when data is available. The triangles show concentrations of total mRNA transcripts in 10^9 transcripts per liter. Only two independent latitudes matched up (< 1° latitude) with metatranscriptome sampling for this cruise. Linear regressions of (C) functionally-annotated, and (D) total mRNA transcript for Dinoflagellata (pink), and the other combined taxonomies (black). Samples are indicated by triangles and colored by taxonomy. Intercepts, slopes and R^2 values (when available) are given in Table 1.

Table S1. Carbon to volume relationships for diatoms and other protist plankton.

Data	log *a*	*b*
Protist plankton*	-0.665	0.939
Protist plankton <3,000 μm	-0.583	0.860
Diatoms	-0.541	0.811
Diatoms >3,000 μm	-0.933	0.881

*Excluding diatoms. Data are obtained from Menden-Deuer and Lessard, 2000 [12].
The equation log pgC cell^{-1} = log *a* + *b* x log V (μm^3) was used to determine cellular carbon content from biovolume, distinguishing between Diatoms (Bacillariophyta; <3000 μm^3 and >3000 μm^3), and 'Protist plankton' (Dinoflagellata, Ciliophora, Haptophyta and Dictyochophyceae; <3000 μm^3 and >3000 μm^3).

Table S2. Single cell transcriptome-derived estimations of mRNA molecules per cell and per C biomass.

Species	Cell diameter (μm)	Carbon quota (pg C per cell)	Estimated mRNA (molecules per cell)	10^9 mRNA molecules per 1 μg C	10^9 transcripts per μg C (1 ~ 50)
Prymnesium parvum (Haptophyta)	8	32	4800	0.15	0.15 ~ 7.5
Karlodinium veneficum (Dinoflagellate)	15	162	51000	0.31	0.31 ~ 15.79

Cell diameter and estimated mRNA molecules per cell are derived from Liu *et al.,* 2017 [13]. Transcript concentrations are estimated assuming 1-50 transcripts for each mRNA molecule, as in Marinov *et al.*, 2014 [3].

References (numbering consistent with primary manuscript):

3. Marinov GK et al. From single-cell to cell-pool transcriptomes: Stochasticity in gene expression and RNA splicing. Genome Res 2014;24:496–510. https://doi.org/10.1101/gr.161034.113
6. Juranek LW et al. The Importance of the Phytoplankton “Middle Class” to Ocean Net Community Production. Global Biogeochem Cycles 2020;34:e2020GB006702. https://doi.org/10.1029/2020GB006702
12. Menden-Deuer S, Lessard EJ. Carbon to volume relationships for dinoflagellates, diatoms, and other protist plankton. Limnol Oceanogr 2000;45:569–579. https://doi.org/10.4319/lo.2000.45.3.0569
13. Liu Z et al. Single-cell transcriptomics of small microbial eukaryotes: limitations and potential. ISME J 2017;11:1282–1285. https://doi.org/10.1038/ismej.2016.190
